# An unusual complication of kidney biopsy: a case report

**DOI:** 10.1186/s13256-023-04338-6

**Published:** 2024-02-03

**Authors:** Ákos Pethő, Attila Fintha, Magdolna Kardos

**Affiliations:** 1 Department of Internal Medicine and Oncology, Faculty of Medicine, Semmelweis University, Budapest, Hungary; 2 Faculty of Medicine, Department of Pathology and Cancer Research, Semmelweis University, Budapest, Hungary; 3 Institute of Pathology, Forensic and Insurance Medicine, Semmelweis University, Budapest, Hungary

**Keywords:** Case report, Kidney biopsy, Bleeding, Autoimmune disease, Scleroderma renal crisis

## Abstract

**Background:**

The kidney biopsy is a routine procedure. Once an indication has been established, the benefit–risk balance may be considered. Sometimes, even with effective treatment, a severe complication may develop.

**Case presentation:**

We present the case of a Caucasian 20-year-old young woman admitted to investigating and treating acute kidney injury. Renal involvement was characterized by kidney damage requiring hemodialysis treatment, positive immunologic testing, 0.5 g/day proteinuria, and microscopic hematuria. Contraindications were excluded, so an ultrasound-guided kidney biopsy was performed. To reduce the bleeding complication, Octostim (desmopressin) was administered. There were no direct complications following the kidney biopsy, so we continued the immunosuppressive treatment. Histologically founded thrombotic microangiopathy. However, 1 week later, severe bleeding developed with the need for urgent surgical left kidney removal.

**Conclusion:**

Kidney biopsy can be considered a routine procedure, and various bleeding episodes are most common in terms of complications, the detection of which is essential. Delayed bleeding complications are rare and can be caused by minor injuries. Our young patient had no injury during the hospitalization. We hypothesized that the developed serious and delayed bleeding complication resulted from effective immunosuppressive treatment. To the best of our knowledge, this is the first such case to date. However, renal biopsy in the case of thrombotic microangiopathy requires caution.

## Background

Kidney biopsy and histological diagnosis became the gold standard in routine nephrology care from far away 70 years ago [1]. A nephrologist or any other physician, for example, a radiologist, could perform the percutaneous kidney biopsy. The first percutaneous technique was described in the year 1951 [2]. The use of real-time ultrasound and automated biopsy needles has simplified and improved the success and safety of this procedure [3]. During percutaneous kidney biopsy procedures guided by ultrasound or computed tomography (CT), it is imperative to take measures to minimize the risk of bleeding and other complications. An ultrasound examination can provide a more precise visualization of the vascularization of the kidney(s), which can aid in determining the optimal location for the intervention. By utilizing this imaging modality, clinicians can ensure that the biopsy is performed with the utmost accuracy and safety [4]. Histopathology has significantly advanced in recent years by introducing new and innovative techniques. These techniques include the use of thin serial sections, special stains, immunofluorescence, and electron microscopy [5].

The kidney biopsy is an invasive procedure with its own potential risks. When a definitive diagnosis is critical for determining the appropriate treatment or gaining valuable insights into the progression and prognosis of a disease, it is prudent to recommend a kidney biopsy to obtain the necessary kidney tissue. This procedure enables a histopathological examination of the tissue, which can provide vital information regarding the underlying disease process. A kidney biopsy is performed almost under local anesthesia. Therefore, it is recommended to obtain a kidney biopsy when a definitive diagnosis is necessary for managing or understanding the progression of a disease. Bleeding is the most common complication [6–9]. As a known side effect, the hemoglobin levels typically decrease in almost all cases after a percutaneous kidney biopsy. However, it is challenging to determine the overall frequency of bleeding due to the inconsistent definitions and diagnoses of bleeding across various published studies. Suffering from a considerable bleeding complication can lead to a substantial alteration in medical procedures. This may result in considerable discomfort, prolonged hospitalization, urinary blockage, need for blood transfusions, interventions, and surgical operations [10]. The risk for bleeding tends to worsen in younger patients, females, and patients with prolonged partial thromboplastin times. In addition, bleeding is worse in patients with elevated systolic and diastolic blood pressures. Bleeding can also be minimized using injectable gel foam at the time of biopsy, a technique often used by interventional radiologists. An arteriovenous fistula occurs after 0.5–10% of biopsies but is usually asymptomatic. Nephrectomy resulting from a kidney biopsy is rare, with a reported frequency of < 0.5% [4]. The risk of death from a biopsy is believed to be < 0.1% in most clinical practices [4]. Retrospective studies show that delayed bleeding after kidney biopsy is rare, and multifactorial factors play a role in its development. Thus, the exact cause could not be determined [11]. There seems to be no increased incidence of severe bleeding during renal biopsy performed with other indications [12]. A recent publication detailing the analysis of a substantial sample of patient data has confirmed the previously reported findings that incidences of severe bleeding complications are exceedingly rare. These latest data reinforce the conclusion that the likelihood of severe bleeding complications is minimal [13]. Complications are more frequently observed in hospitalized patients with acute kidney infections (AKIs) who undergo kidney biopsies [14]. Beyond all this, the presence of infection and chronic disease can worsen the prognosis of this quite risky procedure [15, 16]. Although an autoimmune antibody test can be useful in the diagnosis of autoimmune diseases, a kidney biopsy remains a necessary procedure to confirm the diagnosis. While autoimmune antibody test can provide valuable information, they are not always conclusive and may produce false results. Therefore, a kidney biopsy is still considered the gold standard for confirming the presence of autoimmune diseases and should be considered an essential component of the diagnostic process [17–19].

According to the literature, delayed bleeds may result from minor traumas, whereas severe bleeds occur after discharge. Our case report presents an unexpected late serious bleeding complication that developed one week after the kidney biopsy. We hypothesized that this severe and delayed bleeding complication developed as a result of the effective immunosuppressive and complex treatment of our patient. To the best of our knowledge, this is the first such case to date.

## Case report

The medical history of a 20-year-old Caucasian female patient includes the diagnosis of juvenile rheumatoid arthritis, first documented at the age of eight. At 12 years, the patient could stop azathioprine and methylprednisolone therapy due to complete clinical remission. In May 2017, when she was 20 years old, she developed facial edema and Raynaud’s phenomenon after a viral respiratory infection, following 8 years of clinical remission. Two months later, she was admitted to a county hospital due to pericarditis, pleuritis, and ascites. Anemia, leukopenia, and moderate proteinuria were seen in her laboratory results. Her kidney function and estimated glomerular filtration rate (eGFR) were normal at this time. In repeated blood tests, the platelet count was normal in all cases. Antinuclear antibody (ANA) positivity, high anti-Smith antibody (SM), anti-U1-ribonucleoprotein antibodies (anti-U1-RNP), and low complement levels were found with immune serology testing. Following the series of tests conducted, the patient was diagnosed with systemic lupus erythematosus. Nevertheless, azathioprine and methylprednisolone therapy were restarted, and her clinical symptoms definitely improved. Three weeks later, she presented to the emergency department due to severe suffocation. With laboratory testing, she has found severe acute kidney injury. Due to possible autoimmune origin, a high dose of methylprednisolone treatment (intravenously 500 mg/day) was initiated, and she was transmitted to the intensive care unit. Immunological evaluation verified scleroderma-induced renal crisis in the background of the acute kidney injury. The high dose of methylprednisolone therapy was aborted, and bosentan treatment was initiated. One week later, severe pericardial effusion developed, so the patient was transferred to a cardiac surgery center. During the cardiology hospitalization, she underwent pericardiocentesis, and 1200 ml pericardial fluid was removed. The pericardial fluid seemed transudate and did not contain blood or signs of infection. Her kidney function was worsened; her eGFR reached 8.9 ml/minute/1.73 m^2^, and oliguria developed.

She was admitted to our clinic for the first time on 22 August 2017. From the physical status of the patient, normotension could be highlighted. We started regular hemodialysis treatment through an internal jugular vein hemodialysis catheter. Urine dipstick testing found hematuria and proteinuria; automated sediment revealed leukocytes and erythrocytes but no bacteria. Dysmorphic red blood cells were diagnosed in the urine sediment under a light microscope. Immunologic testing showed ANA positivity, soluble substance A (SSA) of 11 U, soluble substance B (SSB) of 7 U, salivary cortisol level (SCL) of 4 U, Jo1 of 13 U, U1-RNP of 128 U (high), SM of 160 U (high), dsDNA of 28 IU/mL (high), anti-C1q of 53 U (high), complement-3 of 0.62 g/l (low), and complement-4 of 0.06 g/l (low). Based on the results of the immunological tests and the course of the disease, in addition to the renal crisis of scleroderma, the manifestation of Systemic lupus erythematosus (SLE) could not be excluded, so a kidney biopsy was indicated. Before performing the kidney biopsy, we checked the most important and necessary lab results (hemoglobin of 109 g/l, platelet count of 262 Giga/l, prothrombin of 110%, prothrombin time of 8.5 seconds, international normalized ratio (INR) of 0.95, and activated partial thromboplastin time (APTI) of 36.8 seconds). Based on the normal blood coagulation parameters, there were no contraindications for performing the kidney biopsy.

**Fig. 1:**
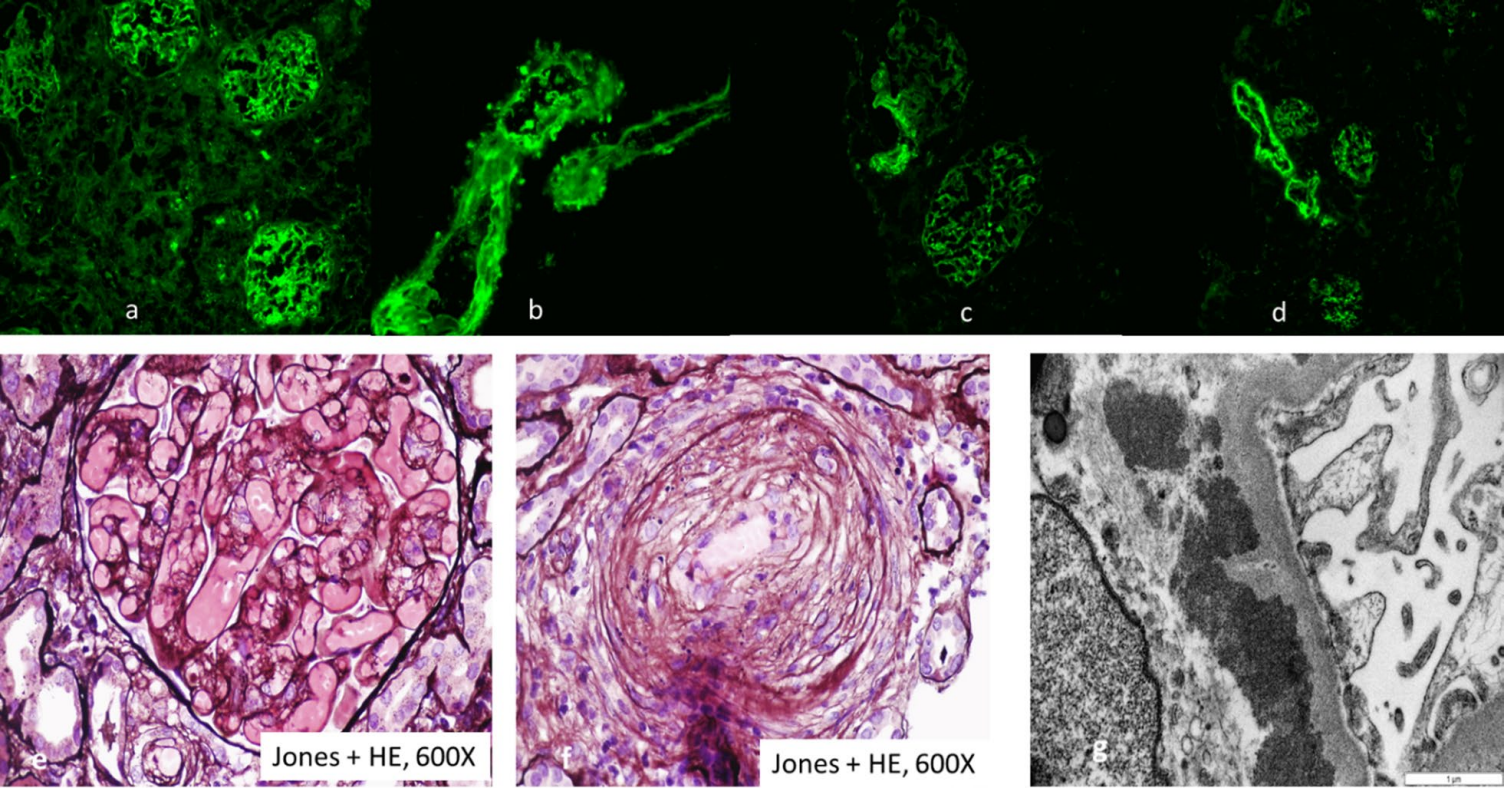
Direct immunofluorescence reaction with fluorescein isothiocyanate (FITC)-labeled anti-IgG (**a**), direct immunofluorescence reaction with FITC-labeled anti-IgM (**b**), direct immunofluorescence reaction with FITC-labeled complement 3 (**c**), direct immunofluorescence reaction with FITC-labeled anti-C1q (**d**), thrombosed capillary loops (**e**), characteristic “onion leaf” lesion in the smaller arterioles (**f**), and electron microscopic examination: subendothelial located electron-dense deposits in the glomerular loop (**g**)

**Fig. 2:**
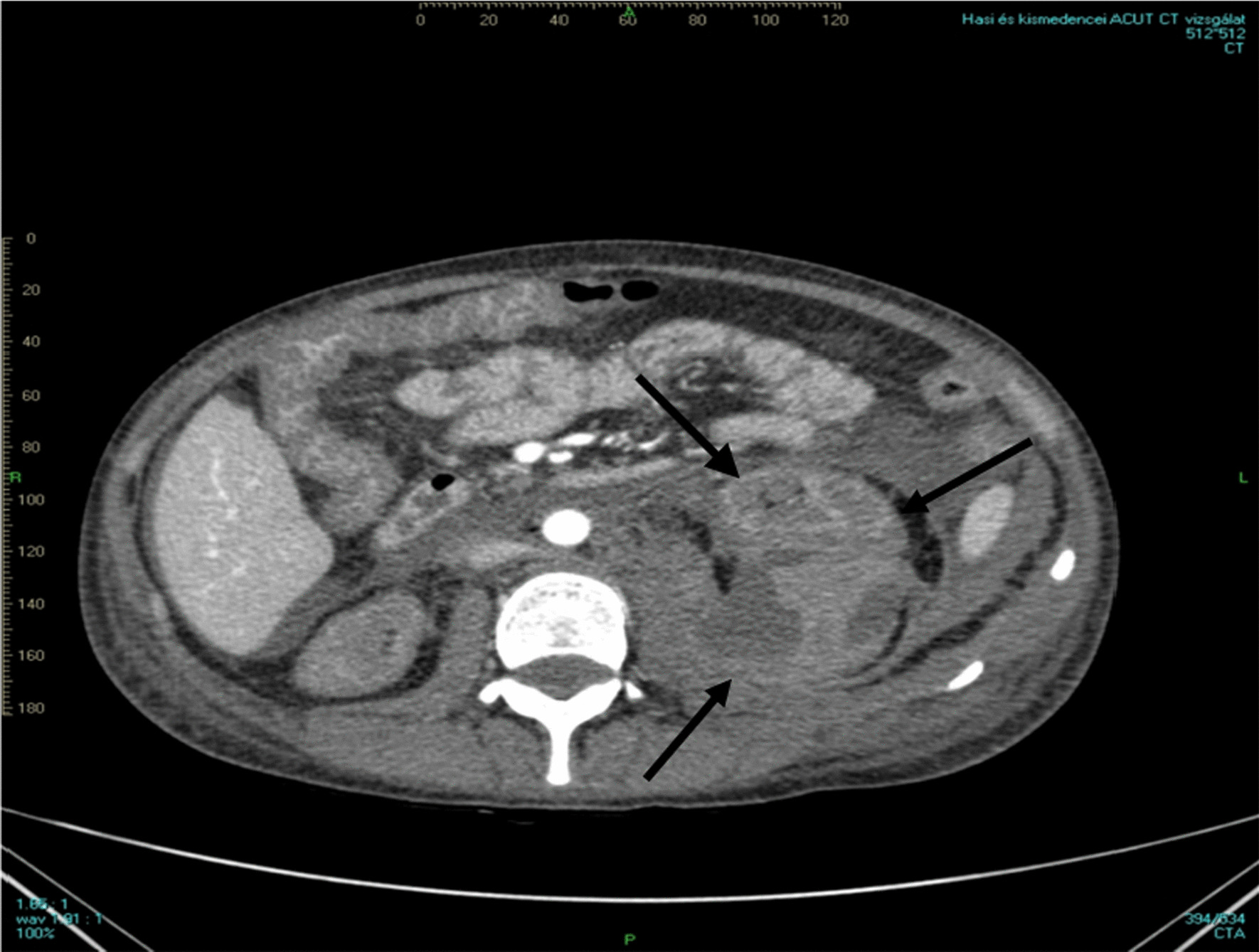
Computed tomography scan shows severe left sided perirenal bleeding with a large hematoma (arrows)

**Fig. 3:**
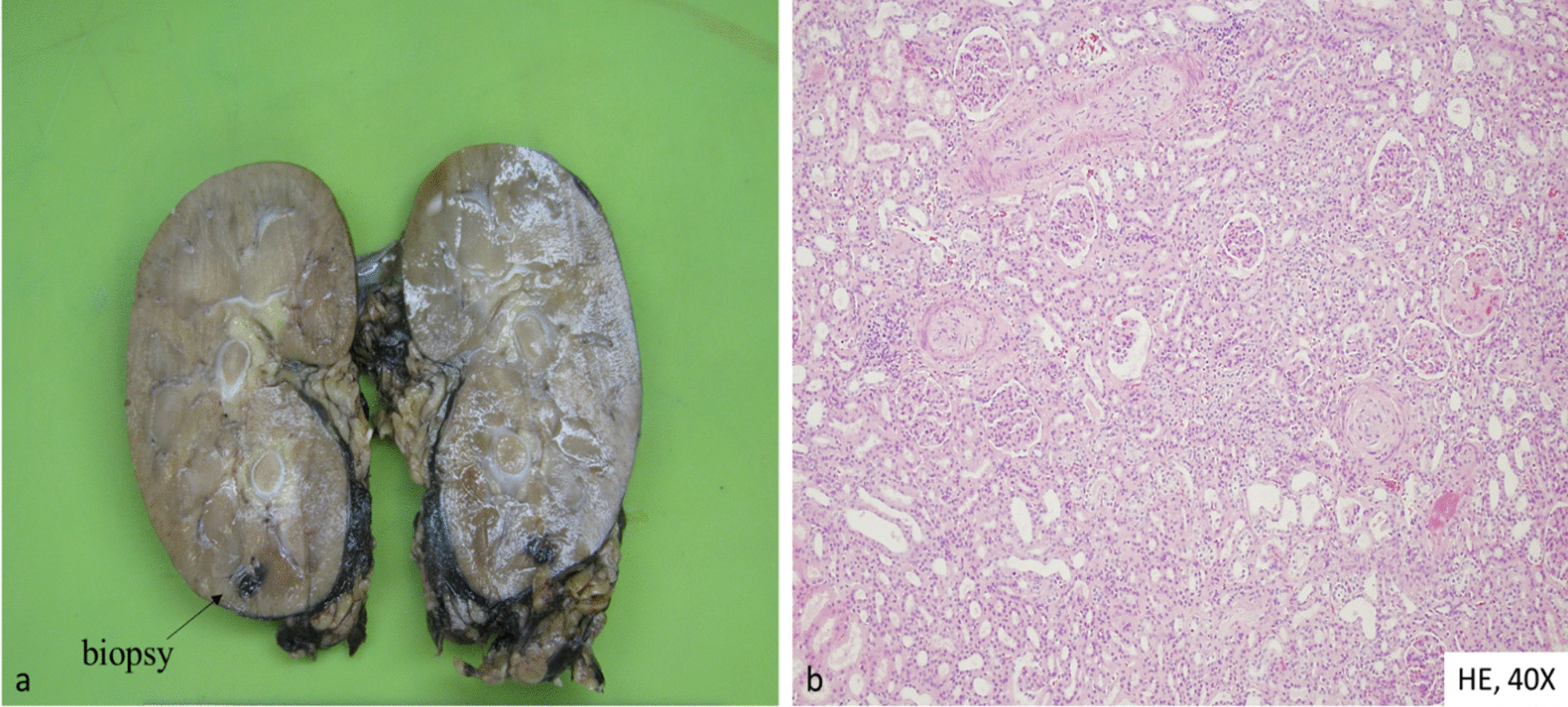
The removed left-sided kidney with the marked kidney biopsy area (**a**); the histological picture was the same as described previously in the biopsy findings (**b**)

**Fig. 4:**
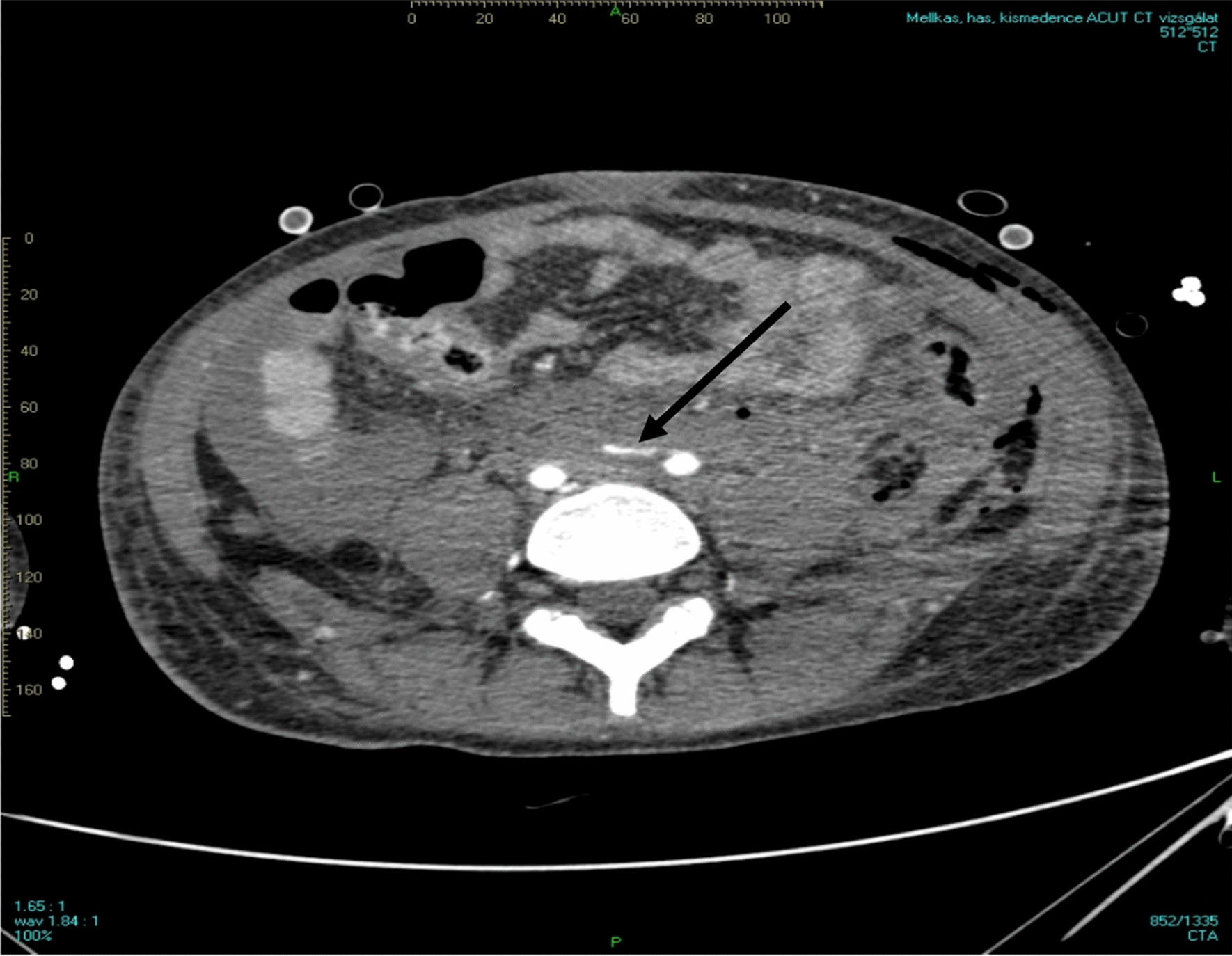
The computed tomography shows that the left-sided arteria iliaca had a radiocontrast extravasation due to artery rupture (arrow)

**Fig. 5:**
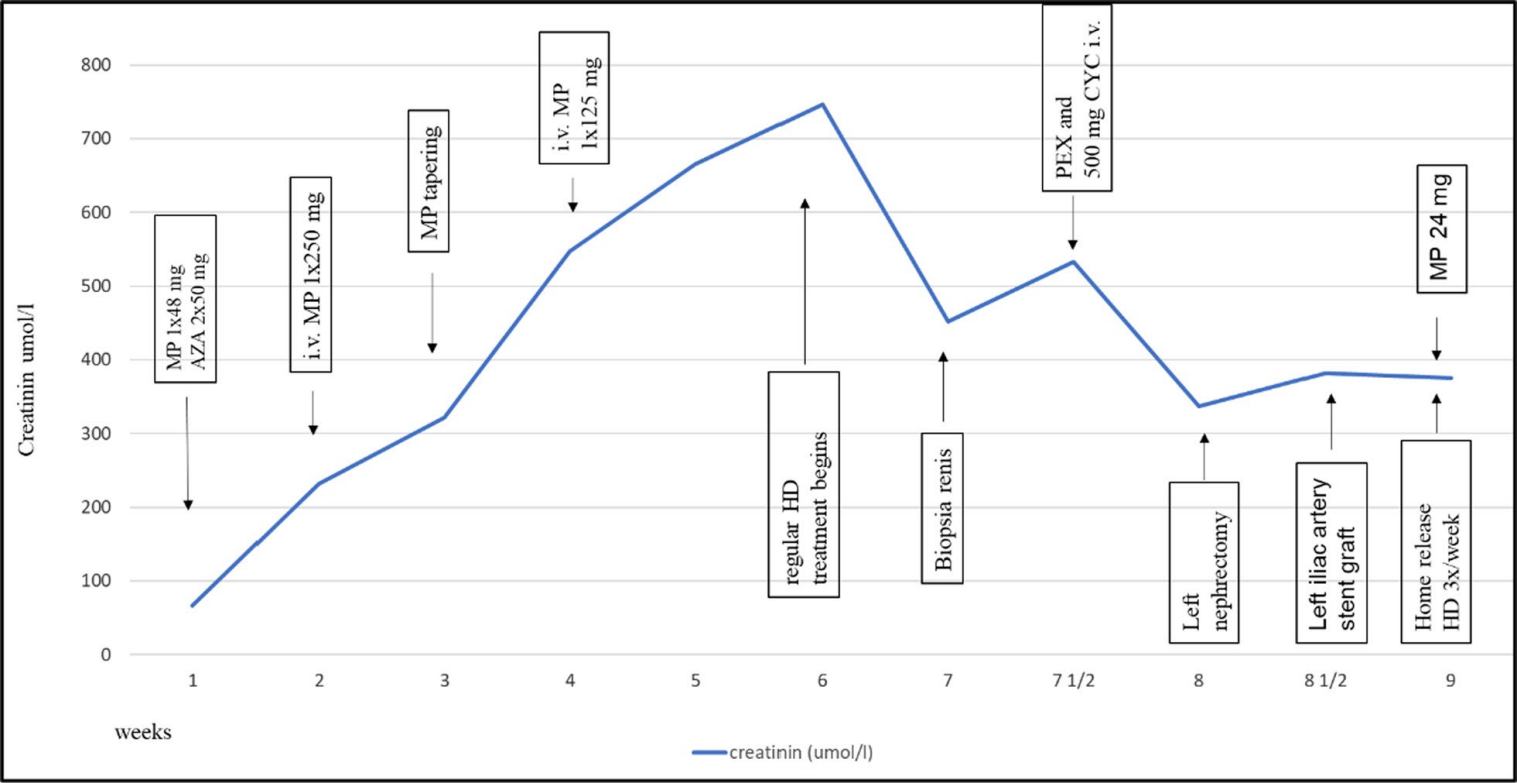
Summarizes the treatment of our patient and the interventions performed. AZA, azathioprine; CYC, cyclophosphamide; HD, hemodialysis; iv, intravenous; mg, milligram; MP, methylprednisolone; PEX, plasma exchange treatment

Three days after admission, we performed a percutaneous and ultrasound-guided biopsy on the lower pole of the left kidney with two punctures. To minimize any probable bleeding complications, we administered 30 ugs (micrograms) of Octostim (desmopressin) intravenously before the procedure. On the following day of the kidney biopsy, an ultrasound examination revealed no signs of bleeding complications. Based on the pathologist’s report, the picture was consistent with scleroderma renal crisis and mixed connective tissue disease (Fig. 1). The pathologist described the typical immunocomplex depositions with the thrombotic microangiopathy and the typical “onion leaf” lesions in the smaller arterioles. Based on the established diagnosis, we determined the course of immunosuppressive treatment. We administered a single dose of 500 mg cyclophosphamide intravenously and performed a single plasma exchange treatment with 2500 ml of 5% human albumin substitution. She remained dialysis dependent. On the sixth day of the kidney biopsy, she developed severe clinical signs of acute bleeding complication (Fig. 2) and was transferred to the intensive care unit. Because of life-threatening anemia (hemoglobin of 26 g/l), she got six doses of blood transfusion, and the left kidney was surgically removed in the operating room to evacuate the hematoma (Fig. 3). Following the completion of the life-saving intervention, further continuous bleeding was detected, and further surgery was indicated. An angiographic computed tomography (CT) scan was performed to localize the origin of the bleeding. The angiographic CT confirmed a potential injury to the left iliac artery (Fig. 4). A vascular surgeon made an iliac artery stent implantation. After the intervention, we did not experience any clinical symptoms indicating further bleeding. After 15 days later of the admission, the patient returned to our clinic in stable condition. Our patient was still anuric and dialysis dependent. She was discharged with regular hemodialysis treatment after 17 days of the first admission. The management of our patient and the interventions that were implemented have been summarized in Fig. 5.

## Discussion

Ultrasound- or CT-guided percutaneous kidney biopsy performed for diagnostic purposes is considered safe. Based on literature data, serious complications occur very rarely [13]. The indication for performing a kidney biopsy is well-defined in several guidelines. It should be clearly performed in the case of unexplained kidney symptoms and kidney damage if no contraindications have arisen [4, 20, 21]. A kidney biopsy was necessary in the case we presented, as the exact diagnosis was unclear based on the tests performed and the patient’s medical history. The immunological results suggested the existence of SLE, which was supported by proteinuria and microscopic hematuria. The clinical picture was complicated by the fact that the renal function of the normotensive patient progressively deteriorated in addition to high-dose intravenous methylprednisolone. Furthermore, the normal count of platelets in the peripheral blood did not support the suspicion of thrombotic microangiopathy. However, a kidney biopsy was necessary.

Scleroderma, or systemic sclerosis (SSc), is a rheumatic illness that impacts the immune system, causing skin fibrosis and harm to internal organs and blood vessels. Although rare, SSc can severely harm an individual’s well-being and may even result in fatality [22]. The therapeutic approach varies depending on whether the sclerosis is local or systemic. On the other hand, scleroderma is a chronic connective tissue disease that is diverse in nature. It is marked by abnormal immune responses, vascular damage, and excessive collagen deposition, leading to fibrosis [23]. In addition, scleroderma is very heterogeneous, leading to significant challenges in treating the disease. The treatment options available for scleroderma mainly focus on reducing inflammation, fibrosis, and issues with blood vessels. They include topical and systemic treatments, such as glucocorticoids, immunosuppressants, biological agents, small molecule compounds, and hematopoietic stem cell transplantation (HSCT). Local therapies involve using local immunomodulators, phototherapy, fat grafting, and surgery [24]. It is essential to be aware that a scleroderma renal crisis (SRC) is a rare but severe complication, affecting up to 15% of patients with systemic sclerosis. Those affected typically have rapidly progressive, diffuse cutaneous systematic sclerosis within the first 3–5 years after experiencing a non-Raynaud sign or symptom. Promptly seeking medical attention is crucial if we suspect we are experiencing symptoms. The SRC is characterized by an acute, usually symptomatic increase in blood pressure, a rise in serum creatinine levels, oliguria, and thrombotic microangiopathy in about 50% of patients [25]. Angiotensin-converting-enzyme inhibitor (ACEi) therapy has significantly improved renal outcomes. Various studies have shown that complement pathways are more active in SSc, and there have been cases where eculizumab treatment has been successful after ACEi and therapeutic plasma exchange failed to produce a serological response. The endothelin-1 blockade is a potential target and therapeutic strategy in SSc-caused vasculopathy [26]. The primary treatment is administering ACEis for SRC, but consuming ACEis before the onset of SRC can elevate the risk of developing SRC and worsen the prognosis, specifically in patients with progressive SSc or systemic sclerosis-related renal vasculopathy [27]. Rarely, patients with SRC may be normotensive on presentation. These patients have poorer prognoses and higher mortality rates than those with hypertensive SRC [28]. The delayed diagnosis and treatment of normotensive SRC contribute to its insidious course. Additionally, current treatment modalities appear less effective in normotensive patients [29]. According to the kidney biopsy, there was evidence of vascular thrombosis, severe glomerular ischemic collapse, and peritubular capillary C4d deposits in the SRC kidney biopsies. This indicates a higher likelihood of failure to recover renal function [30]. It is important to note that patients with SSc taking corticosteroids and having normal blood pressure are at a greater risk for SRC [31, 32]. Although a conclusive connection has not been established, it is suggested that corticosteroids may impede the generation of prostacyclin while increasing the activity of angiotensin enzymes. Regrettably, numerous cases of normotensive SRC have led to a devastating outcome as per-reported incidents [33]. Glucocorticoids are widely used in various autoimmune diseases but have a controversial role in managing SSc.

For those patients, it is advisable to stick to low or medium doses, typically not exceeding 15 mg of glucocorticoids per day. This is to prevent the possibility of SRC, which could occur if higher doses were administered [34]. Our young patient experienced the same problem. She was issued a high dose of methylprednisolone, which resulted in her SRC. The primary diagnosis was mixed connective tissue disease (MCTD), based on her immunologic testing and clinical signs. The anti-U1RNP antibody is generally known as a serological marker for MCTD [35]. It is worth noting that this antibody cannot be considered a conclusive immunologic marker for SSc. Therefore, relying solely on its presence may cause delays in making accurate diagnoses [36]. The kidney biopsy results confirmed thrombotic microangiopathy, which explains why no complications were observed after the intervention. Patients suffering from SSc have a higher risk for organ bleeding than the general population [37]. We hypothesized that the immunosuppressive treatment had a positive effect, as it caused the kidney capillaries to open. Our hypothesis is strengthened by the fact that no apparent cause for the development of serious delayed bleeding complications could be proven in the cases reported so far. Furthermore, our young patient was under continuous medical observation, so this delayed severe complication occurred during his hospitalization. Of course, this is also a weakness of our hypothesis, because we cannot prove this by comparing it with a control group. It is essential to consider the potential role of heparin-derivates in dialysis treatment and their impact on bleeding. However, it is noteworthy that in the case of our patient, who was dialysis dependent, bleeding would have manifested much earlier if this was the cause. It is essential to evaluate all possible factors that could cause bleeding in dialysis patients and to take prompt action to prevent and manage such complications. Furthermore, a traumatic event or serious injury could be ruled out in the background of the severe bleeding complications that developed in our patient.

## Conclusion

In many cases, a kidney biopsy is an essential diagnostic intervention. Despite the appropriate care, practice, and precautions, we must count on the occurrence of complications. We reported a serious bleeding issue that occurred 6 days after a kidney biopsy during hospitalization. There is an increased risk of bleeding in cases of thrombotic microangiopathy during kidney biopsies. We recommend that caution be exercised when performing kidney biopsies in patients with SSc; after the intervention, careful monitoring of the patient is recommended. This may be remarkably advisable when specific immunosuppressive therapy is used in treatment. Our research hypothesis posits that in autoimmune diseases affecting small vessels, a potential for bleeding complications may arise following a kidney biopsy. It is imperative to consider these complications, which may manifest at a later stage, to ensure the safety and well-being of the patient. We recommend that healthcare professionals remain vigilant of these potential postbiopsy complications.

## Data Availability

Data sharing does not apply to this article as no datasets were generated or analyzed during the current study.
